# Explainable AI: A Neurally-Inspired Decision Stack Framework

**DOI:** 10.3390/biomimetics7030127

**Published:** 2022-09-09

**Authors:** Muhammad Salar Khan, Mehdi Nayebpour, Meng-Hao Li, Hadi El-Amine, Naoru Koizumi, James L. Olds

**Affiliations:** 1 Schar School of Policy and Government, George Mason University, Arlington, VA 22201, USA; 2 Volgenau School of Engineering, George Mason University, Fairfax, VA 22030, USA

**Keywords:** explainable AI, interpretable AI, AI, decision stack, neurally inspired

## Abstract

European law now requires AI to be explainable in the context of adverse decisions affecting the European Union (EU) citizens. At the same time, we expect increasing instances of AI failure as it operates on imperfect data. This paper puts forward a neurally inspired theoretical framework called “decision stacks” that can provide a way forward in research to develop Explainable Artificial Intelligence (X-AI). By leveraging findings from the finest memory systems in biological brains, the decision stack framework operationalizes the definition of explainability. It then proposes a test that can potentially reveal how a given AI decision was made.

## 1. Background

The recent crashes of two Boeing 737-Max commercial airliners have raised important questions about an embedded computational system (MCAS), which was installed to make the new 737 models feel more like the older models for human pilots [1]. Among the key issues raised is that the human pilots were not informed about the existence of the system and that the system’s “intelligence” was subject to a single point of failure (an angle of attack sensor) [1]. Increasingly, Artificial Intelligence (AI) will play a significant role in such systems, particularly as autonomous machines operate in remote and hostile environments such as space or deep ocean [2]. In that harsh context, when failures occur, it will be essential to precisely assess what went wrong so that the designers can learn from failures. Meanwhile, when such systems make evidence-based decisions, explaining why and how a given decision was made is crucial. European Union law warrants such an explanation as part of the “Right to Explanation” enacted in 2016, mainly in the context of adverse decisions affecting citizens.

Modern AI systems operate on noisy and often uncertain data to make decisions on behalf of humans. When these systems work they are of great utility allowing for, among other things, self-driving cars and autonomous robots that operate in hostile environments. Beyond utility, these systems can also engage in self-teaching modes that allow them to excel beyond human capabilities at games like chess and Go [3,4,5].

However, as with human intelligence, sometimes AI fails to deliver. A well-known instance of such a failure is a Tesla Model S that was involved in a fatal crash while the car was in “self-driving mode” due to inaccurate feature extraction and intelligent comprehension of a white-colored truck by the AI [6]. The failure of AI is not surprising. Intelligence is the act of making decisions based on uncertainty. This fact differentiates AI from non-intelligent decision systems based on the flow-chart design, as in most computer programs [7]. For human beings, such failures are required for many kinds of learning during childhood and adulthood. Most machine learning (ML) AI algorithms also depend on a “training phase” whereby the artifact is instructed on a human-labeled dataset and learns from its failures before being allowed to operate in the “wild” on non-labeled data [8]. Therefore, it is understandable that, despite training, both humans and AI might mislabel a new instance of data that had never been seen or used before.

In the case of human intelligence, only recently has neuroscience offered a clearer picture of the cellular basis of learning and memory [9]. Furthermore, neuroscience provides evidence of a concrete hierarchy with the human body–brain system at the top and neuronal synapses at the base, allowing for a framework for explaining human decisions and their concomitant failures [10]. However, for AI, the explanation of why failures occur is not readily explainable [11]. This is in spite of European Union law requiring that such explanative AI be available to EU citizens to protect them from potential adverse effects of AI-based decisions such as the denial of credit [12,13].

Explainable AI (X-AI) is an artificial intelligence system capable of describing its purpose, rationale, and decision-making process in a manner that the average person can understand [14]. Here, we propose to advance the idea of a *decision stack* as a framework for explaining AI decisions, including failures. The term is beneficial as it reflects the idea that explanations must cross different levels of an organization in terms of complexity and abstraction [15]. In the next section, we will briefly lay out the literature that defines the field of X-AI and describe how our theoretical model fits in the field to explore a new way of looking at X-AI.

## 2. Explainable AI

The problem of explaining the outcome of a decision process is not new [16,17]. What makes X-AI fundamentally different is the added scale and dimensionality in modern AI decision systems compared to traditional decision trees or regression models [18]. This is where the problem of explainability becomes critical. As Paudyal puts it, X-AI is not an artificial intelligence system that can explain itself, but it is what we can try to interpret from the outcomes of an AI system based on our limited understanding of a process [19]. That is why some researchers use the word explainable AI interchangeably with interpretable AI. In other terms, how can we interpret an AI outcome to satisfy a specific question regarding the process that produced the outcome? It is important to note that in the attempt to understand the explainability of AI, we are not interested alone in the accuracy of the outcome of AI per se. In other words, not only do we want to explain why and how a wrong decision was made, but we also want to know why and how a correct decision was made.

Our interest in the process of decision arrival stems from the fact that a decision outcome could be true, but an AI system’s process to reach that outcome might not be desirable based on our values. For example, an AI classifier that was designed to detect wolves among dogs, although it had perfect accuracy, based its decision on the presence of snow in the background of pictures with wolves [20]. In another instance, a law enforcement AI model designed to predict the risk of repeating criminal activity was found to have a strong racial bias [21]. AI systems can take shortcuts to arrive at a decision; however, these shortcuts are not always desirable. Therefore, the explainability of an AI system is separate from the accuracy of that decision system. This makes the attempt to develop an X-AI framework even more vital.

Various critical voices in the AI literature fundamentally challenge the concept of X-AI. We summarize these criticisms here in two groups. The first group believes AI is essentially too complex to be explainable. For example, researchers in the first group argue that the most popular AI systems have close to 100 million parameters, making it impossible to explain any specific outcome objectively; thus, this group believes that X-AI is a pointless endeavor [19].

The second group believes X-AI is possible but points out the challenge of the performance–explainability tradeoff. This tradeoff refers to the theory that in deep machine learning algorithms, more inputs and more hidden layers in a prediction model increase the accuracy of a model while interpreting the outputs becomes more challenging [19]. Thus, they warn us that emphasizing too much on explainability might significantly damage the performance of a model.

In line with the first group’s criticism, we acknowledge the complexity of AI. However, like most researchers, we believe that the question is not whether we can explain complex AI or not, but to what extent can we explain AI, what is a satisfactory explanation, and for what problem is the explanation needed [22]. As for criticism from the second group, we believe that line of criticism is concerned with the matter of design, i.e., how to implement a sufficient level of explainability to the design of an AI system. In our case, we focus on the problem of explaining an AI outcome post its design and development. Thus, the performance–explainability tradeoff is irrelevant here.

Explainability is crucial in policymaking and litigation [23]. Policymakers are especially expressing concerns over the emergence of “Blackbox” systems, which challenges the effectiveness of regulations [23]. That is why the “right to an explanation” movement, as mentioned, has been gaining momentum in policy circles, especially in Europe. Concurrently, the past few years have seen a plethora of literature on the importance of X-AI [24,25,26,27]. Parallel to the technical side of X-AI, a separate body of literature has been addressing explainability from the perspective of human rights, social justice, and fairness [28,29,30,31]. Felten has previously surveyed the X-AI literature and explains that any successful X-AI endeavor should help AI systems reach these four goals: transparency, accountability, safety, and fairness [32]. The X-AI framework presented in this paper aims to be used as a foundation for any domain-specific X-AI system to improve its transparency, accountability, safety, and fairness.

One final critical aspect of X-AI that has gained attention in the literature is the issue of evaluating the explainability power of an X-AI framework. Simply, how can we assess if an X-AI framework has adequately explained a decision process to an end-user? Scholars point out to the fact that the final goal of X-AI is eventually to convince a person about the credibility of an AI outcome [22]. This puts the end-user at the heart of X-AI. That is why ‘the power to convince’ is central to most X-AI evaluation frameworks. Hoffman et al. have proposed the most comprehensive guidelines for evaluating X-AI frameworks [25]. They built their guidelines based on the idea that “the property of ‘being an explanation’ is not a property of statements; it is an interaction” [25]. They also note that an explanation depends on end-users needs and their understanding of the AI outcome. Hoffman et al. essentially propose a qualitative effort using satisfaction surveys, questionnaires, mental models, and checklists.

While Hoffman et al. have successfully offered an evaluation model for X-AI frameworks, it relies heavily on end-users subjective understanding and satisfaction. Unfortunately, the literature lacks a universal objective framework that can explain an AI outcome independent of the end-user, i.e., an explanation that can be used in a court of law or to solve a policy problem where objectivity is desired. Our proposed theoretical framework in this paper aims to satisfy this need. However, before we offer our framework, we summarize existing X-AI methods and some of their issues below.

## 3. Current State-of-the-Art Explainable AI Methods and Approaches

**Figure 1:**
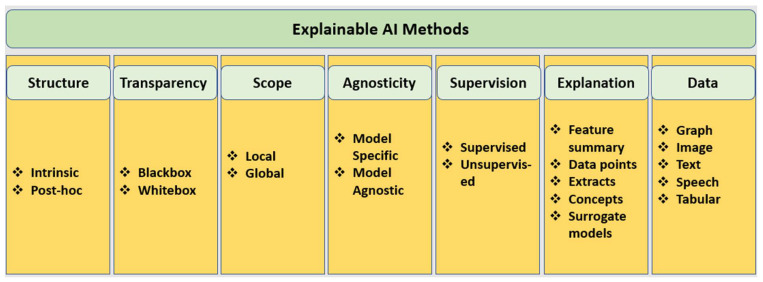
The figure lists various explainable AI methods.

Machine learning interpretable techniques (or X-AI methods) aim to understand ML models’ decisions (predictions) and explain them in human terms to establish trust with stakeholders, including engineers, scientists, consumers (users), and policymakers. The field is not nascent [33]; its early days trace back to the origins of AI research in the development of human ML systems [34]. Since about 2015, there has been a resurgence in X-AI research that parallels the advance in increasing problems of applied ML systems in society. As a result, we have seen a suit of interpretable ML or X-AI methods particularly to untangle deep learning models [34]. One can choose from various ML interpretability techniques (shown in Figure 1) for any use case [34,35,36,37].

Below, we summarize a landscape of the techniques (listed in Figure 1) from the literature based on certain criteria, including structure, design transparency, agnosticity, scope, supervision, explanation type, and data type.

### 3.1. Intrinsic vs. Post-Hoc: The Criterion of Structure

This criterion differentiates whether interpretability is achieved by containing the complexity of ML models, known as intrinsic (aka simple models), or by applying methods that analyze models after training (called post-hoc models) [35,38,39]. Intrinsic models are easily interpretable because of their simple structure [39]. Examples of the intrinsic model are linear regression, logistic regression, decision trees, and k-nearest neighbors. Post-hoc are complex structure models that achieve interpretability after model training [39]. Examples include permutation feature importance and neural networks [40]. Such models take into account changes in the feature or neural space and how these changes affect the outputs.

Generally, intrinsic models return simple interpretable explanations, but they lack in offering high-level predictions for complex problems [34]. On the other hand, post-hoc models perform better on most tasks but are too complex to understand for humans [34]. Neural network models, for instance, have millions of parameters that surpass human capabilities. These post-hoc models necessitate the need to derive human explanations for complex ML models.

### 3.2. Blackbox vs. Whitebox vs. Greybox Approaches: The Criterion of Transparency in Design

This criterion distinguishes based on what we know about the design of a method. A Whitebox approach is more transparent and explainable by design than a Blackbox approach [41]. Examples of Whitebox approaches include simple decision trees, rule-based models, patterns-based models, linear regression models, bayesian networks, and fuzzy cognitive maps [42,43]. Other methods following the Whitebox approach include fuzzy decision trees and fuzzy rules-based models. As opposed to Boolean logic (true/false statements, for instance), such algorithms follow a fuzzy logic (human-like reasoning with statements that could lie in a spectrum of truth/false) and hence take into account an uncertainty underlying analyzed data in explaining the decision [44,45,46,47,48,49].

As opposed to the aforementioned Whitebox methods, deep neural networks and random forests are some examples of Blackbox approaches [41,50]. Blackbox approaches usually contain complex mathematical functions like support-vector machine and neuronal networks; thus, they are generally hard to understand and explain [43]. On the other hand, most Whitebox models can be comprehended by experts as their models are closer to the human language [43].

In the Blackbox approach, we only know the relations between inputs and outputs and the response function to derive explanations [51]. As far as Whitebox approaches, we have access to the model-internal parameters [51]; in other words, we can access weights or gradients of a network. In general, Whitebox approaches are more interpretable but less accurate, whereas Blackbox approaches are more accurate but less interpretable [41].

Finally, there are Greybox approaches as well, which lie in between Blackbox and Whitebox models [41,52]. Examples of such approaches include Local Interpretable Model-agnostic Explanations (LIME) and Interpretable Mimic Learning [41]. In Greybox models, an expert knows when how some part of a system works mathematically (Whitebox) and is uncertain about the others (Blackbox). The Whitebox part of such models is fixed due to the underlying physical structure and constraints, whereas Blackbox part needs to be learned from the data. Greybox models, combining Blackbox and Whitebox features, may acquire the benefits of both, causing an explainable model which could be both accurate and interpretable simultaneously [41].

### 3.3. Local vs. Global: The Criterion of Scope

This criterion distinguishes methods based on whether the scope of the interpretability applies to the whole or part of the model. In local approaches, the scope of interpretability is limited to individual predictions or a small portion of the model prediction space [53,54]. On the other hand, global methods cover the entire model prediction space [53,54]. This is accomplished by aggregating input variables’ ranked contributions towards prediction space (or decision space).

Local approaches provide a larger precision particularly of individual prediction (or a specific decision) but lower recall understanding of model behavior across all examples [54]. On the other hand, global approaches have a higher recall view of the model prediction (i.e., help one comprehend complete decision structure) but lower precision due to aggregations such as medians or means, which obscure individual contributions [54].

Some examples of local approaches are Local Interpretable Model-agnostic Explanations (LIME) [20]. SHapley Additive exPlanations (SHAP) [55], and Individual Conditional Expectation (ICE) [56]. Examples of global approaches include Partial Dependence Plot (PDP) and Accumulated Local Effects (ALE) [56,57].

### 3.4. Model Specific vs. Model Agnostic: The Criterion of Agnosticity

This criterion differentiates XAI methods on the level of agnosticity. Model agnostic methods refer that their X-AI algorithm can be applied to any kind of ML model [54,58]. Such methods do not depend on model internals. Instead, they rely on changes in input features or their values to understand how they influence the outputs of a use model. Examples include SHAP and LIME, which are portable across different model types [20,58]. Conversely, model-specific methods are designed for specific types of ML model [58]. Examples are methods that depend upon some intrinsic parts of model learning methods, such as neural network methods [54,58].

Agnosticity criterion could be a blurry boundary between various methods. One may aggregate the scores of some local model-specific methods (such as integrated gradients or SHAPLY) to revive the entire prediction space employing aggregation operations like averages and medians. Such methods would be termed as hybrid methods [59,60].

### 3.5. Supervision-Based Methods

This criterion distinguishes among methods based on the degree of supervision. Some examples of these methods are AI attribution methods, rationale-based methods, and disentanglement representations. While attribution methods entail an active manipulation of input data (supervised), rationale and disentanglement representations are unsupervised methods in the sense that researchers assume no explicit annotations about input data. Examples of attribution methods include LIME, SHAP, Integrated Gradients, SmoothGrad, Layer-wise Relevance Propagation, and Perturbation methods [61]. In rationale methods, pieces of input texts are extracted to determine a possible justification (Rationale) for prediction [62,63]. Researchers have no say in determining which words should be included in the Rationale [62]. Similarly, in disentanglement representations, latent-variable models learn representations of high-dimensional data in an unsupervised manner [64].

### 3.6. Explanation Type-Based Methods

There is a variety of interpretability methods that further differ in explanation output [35,58]. For instance, techniques such as feature summary return feature statistics, measuring a feature’s proportional contribution to the prediction [65]. Similarly, other returning data points help us better understand the models [66]. Some different approaches help us build simple models around complex ones. Those simple models, called surrogate models, can be used to derive explanations [35]. Finally, other explanation type-based methods extract concepts, decision rules, correlation plots, and other visualizations [67,68,69].

### 3.7. Data Type-Based Methods

Besides all the above criteria, we can further differentiate according to the data type a method can handle [70]. Not all X-AI algorithms can work with all data types. Examples of data types may include graph, image, text/speech, and tabular [35,71,72,73,74].

The above paragraphs provide an overview of the various methods that have already been developed. Readers interested in more details can refer to other established and emerging literature [34,35,36,37,38,70]. Overall, each X-AI method has different guarantees, limitations, and computational requirements in explaining outputs. Nevertheless, these excellent foundational methods help create some model understanding and offer bits of human interpretable understanding. However, there is still so much for the scientists and engineers to understand how the AI implemented a decision while explaining the model decision to the public, policymakers, and regulators.

The methods are not a panacea to all our problems in the X-AI research field [75,76,77]. One issue discussed in the previous section is the interpretability–performance tradeoff [77,78,79]. For the last decade, as researchers and engineers have tried to increase performance or even exceed human-level performance through AI algorithms (for instance, via deep learning algorithms), the effort has costed reduction in the level of explainability.

High-profile ML deployment failures from these Blackbox models offer convincing evidence of the claim made in the preceding paragraph [80,81,82,83,84]. The failures point out that the models, particularly Blackbox (post-hoc) models and methods, are very opaque, uncontestable, exhibit unpredictable behavior, and in some cases reinforce undesirable racial, gender, and demographic biases [85]. All these affect crucial outcomes for the public, engineers, scientists, and policymakers. High-stake settings such as healthcare, criminal justice, and lending have already reported significant harms because of the problems inherent in Blackbox methods [85].

Another issue is the kind of explanation these X-AI methods return. So far, the explanation is incomplete (i.e., still model output is not fully predicted) [86,87]. Similarly, the explanation lacks accuracy. For example, four X-AI methods (including LIME) were deployed to predict a matchstick [86]. In other words, researchers were curious to know what makes an image of a matchstick a matchstick. Is it a matchstick because of the flame or a wooden stick? In their default settings, by changing a single parameter, the methods returned 12 unique explanations suggesting the methods are unstable in prediction [88]. The X-AI methods may also misdiagnose cancer if medical images can get modified in ways unknown to human understanding [89]. In a similar vein, the explanation must be meaningful and understandable. Unfortunately, X-AI methods have yet to make strides to return meaningful explanations. In one instance, deep neural networks mislabeled the image of a lion as a library [90]. All these issues might get resolved once the scientists thoroughly research and understand the intricacies within Blackbox models.

Finally, another vital challenge these methods face is the challenge of internal and external validation. Internal validation demands that we rule out alternative explanations, establish causality direction, and account for simultaneity and selection bias [91]. However, we have seen the internal validation of a method severely challenged by changing the input data (features or other data parameters). For instance, in the famous example of husky vs. wolf, the X-AI method (LIME as developed by Ribeiro et al. [18]) failed to predict a husky on the snow due to a bias in data. As opposed to the training set (a husky on the grass, a wolf in the snow), in the validation set the researchers flipped the usual background in some images (husky on the snow and wolf on the grass). Another instance of failed internal validity is when autonomous vehicles misread (mispredict) a slightly blurred stop sign [81], or, by changing a single pixel on an image makes an AI think a horse is a frog [82], or in the case of medical imaging, X-AI algorithm misclassify a brain tumor [89]. Similarly, AI methods failed to predict a school bus’ right-side-up when the bus was rotated [80], suggesting AI’s brittleness and weak internal validity. All these have repercussions for users.

Unlike the model’s precision and internal mechanics, external validation would demand a successful application of X-AI methods from a small validation dataset to a larger population or on a range of data [91]. This is essential as AI moves from toy lab experiments and returns results on wild data in different circumstances. Existing X-AI methods suffer from external validity concerns and cannot handle all sorts of data. Some methods work with visuals; other techniques take only text and speech. The validity issue is even more problematic when the methods applied to the same data return different predictions, as elaborated in the example of matchstick prediction [86,92]. In the exact matchstick prediction, some researchers increased the sample size from 50 to 800, and the prediction space of heatmaps changed [93], suggesting the X-AI method is not robust when the sample size increases. Such external validity issues will be highly concerning as AI applies to human ML systems in healthcare, legal, defense, and security arenas.

There is an urgent need for an X-AI framework that explains the model and educates the users about AI decisions, building trust among various societal stakeholders. Such a framework will ideally return complete, accurate, consistent, reliable, and meaningful explanations comprehendible in various instances across multiple domains both in the lab and the practical world. In this concept paper, we look to the human brain for inspiration and derive a neurally inspired framework named as decision stack framework. Our goal for the framework is to employ any dataset, mimic human brain circuitry, and generate meaningful understanding, thus disclosing the AI decision Blackbox in its entirety. Furthermore, since the human brain is intelligent, chosen for throughout evolution, and universal [2,94], we believe the framework will be robust and efficient in prediction. In the following paragraphs, we elaborate on the lessons from neuroscience for motivation and explain the non-AI machine decision stacks for comparison before elucidating our neurally inspired framework.

## 4. Existence Proof: The Lessons from Neuroscience

Since the advent of non-invasive human functional brain imaging in the last decades of the 20th century, it has become commonplace to observe the brain correlates of conscious human subjects as they make decisions at a resolution approximately 1000-fold greater in space and time than the neural code itself [14,95,96,97,98,99]. These blurred images of human brains “caught in the act” of making intelligent decisions have been striking, albeit problematic, from the standpoint of reproducibility. Nevertheless, scientists have observed clear localized neural signatures of learning and memory [100,101]. Impressively, AI systems have been trained on such signatures and can correctly label them with appropriate nouns (e.g., cup, banana).

Blurred images of human brains in action have been connected to the lower levels of the human decision stack by numerous animal studies that recorded from individual nerve cells and even individual synapses [102,103]. These animal studies assume that mechanisms of mnemonic function have been conserved across phylogeny. Recent work on human epilepsy patients supports this notion [102,103]. Operationally, neuroscience has coined the word “engram” to represent the cell assembly of neurons that participate in an individual memory [104]. These engrams can be visualized in animal models and correlated precisely to cognitive and behavioral states in the same experimental subject.

**Figure 2:**
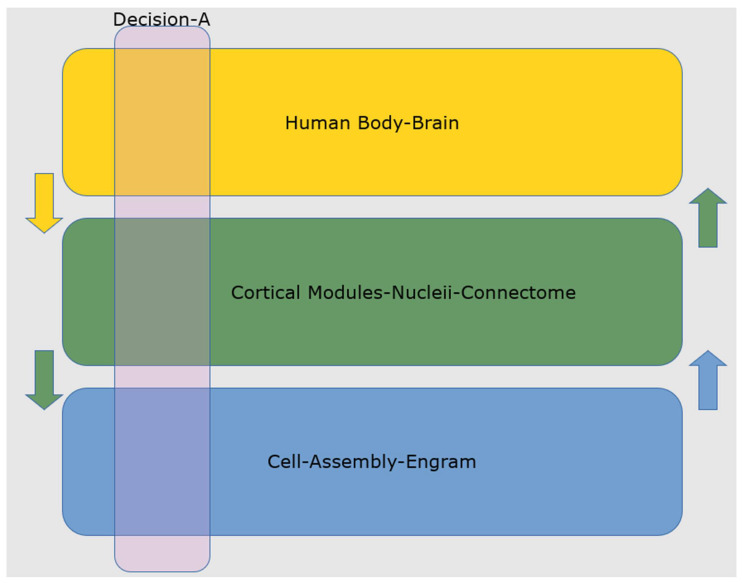
The Human Brain Decision Stack. Primary control flows from the bottom to the top of the stack, albeit with top-down feedback. A set of individual neurons (c. 1000 out of 10^11^) make up a cell assembly representing a perception, concept, memory, or decision. The synchronous behavior of the cell assembly results in specific activations in brain cortical modules and nuclei via short-range and long-range axonal connections, known as the connectome. Those activations produce a cognitive response in the human body-brain, manifesting as decisions and human behaviors.

Most importantly, scientists have developed novel optogenetic techniques that enable experimenter-controlled switching on and off of specific engrams to produce corresponding amnesia and subsequent memory rescue in animal models, including mice [105]. Thus, the base of the decision stack, at the level of the cell assembly, has been connected to the top of the stack, at the level of the brain-body. The intermediate components of the neurobiological decision stack correspond to various cortical modules, brain nuclei, and their associated connections, as illustrated in Figure 2.

The revealing of the animal decision stack allows for a full explanation of animal decisions, as evidenced by the optogenetic studies described above [105]. By extension, such explanatory capability should be available in human subjects, provided that the spatio-temporal resolution of non-invasive functional brain imaging can be extended to the level of the human neural code (engrams). While a problem can explain an intelligence failure in the lowest level of the stack, it might also be explained by a problem above that layer. The entire functioning stack framework is necessary to explain neural intelligence failure.

While the primary flow of control in human brains is from the bottom of the stack to the top, there is an additional phenomenon that adds to the complexity and functionality of the human decision stack: feedback information and control from higher to lower levels. Such feedback is known to optimize the computational efficiency of neural computation. It has been selected for throughout the evolution of brains because of energy constraints, i.e., the human brain operates on about 20 watts of electricity, the same power as a refrigerator light [106].

## 5. Non-AI Machine Decision Stacks

**Figure 3:**
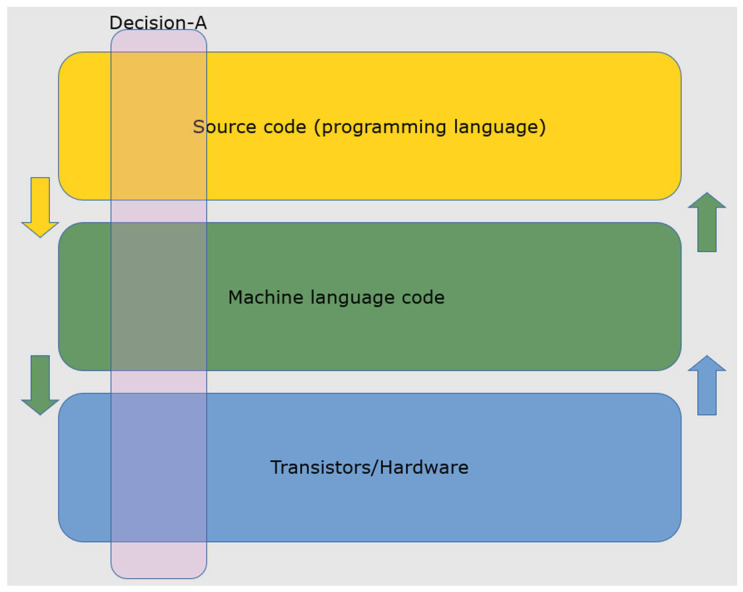
The decision stack for a non-intelligent digital machine. Source code for a computer program is written in a programming language (e.g., Python). The source code describes an algorithm to operate on structured data to produce a decision. For all intents and purposes, the program is deterministic. An interpreter or a compiler translates the source code into machine language, which then instructs the hardware layer to turn on and off electronic components (transistors) to produce a decision that is then translated to the human operators via an interface.

Non-AI failures occur, often manifesting on our electronic devices. When a software engineer debugs a computer program, that process reveals the explanation for failures embedded in the program’s source code. Such debugging has been evolved and engineered over the years to facilitate the central roles of human beings in debugging. While source code resembles a written language like English, it must be translated into machine code to execute on a digital computer. Machine code ultimately becomes the binary sequence of 1′s and 0′s that drive the transistors that populate the Complementary Metal–Oxide–Semiconductor (CMOS) circuitry of extant devices. Programing languages and debugging routines are human-engineered tools for explaining non-AI machine failures in digital computers. However, it is the framework of the machine decision stack illustrated in Figure 3 that enables the full explanation of such failures. Source code bugs must be compiled before they produce failure. The failure eventually manifests in the incorrect behavior of CMOS electronics.

Reviewing the machine code to derive the explanation of a program failure is an alternative to current software debugging routines. However, this would be very difficult, as evidenced by the slow speed of debugging the earliest digital computers that used machine language [107].

Since the machine code drives billions of transistors in modern computers, the *Gedanken* experiment of reading out each of their states to explain failure would be daunting. In contrast to the human brain, most explanation for failure comes from observing the system at the top, not the bottom. We observe this in a web browser that freezes on our computer desktop. The explanation is usually found in error in the source code written by a human that has propagated to the machine code level and then to transistor hardware. Hardware malfunctions are equally capable of explaining failure modes. For this reason, information flows in both directions in the machine decision stack found in modern devices, as depicted in Figure 3. For example, when the transistors inside the CPU of a modern computer become too hot, they can transmit that “distress signal” upwards to either slow down or interrupt the functional execution of a program. Furthermore, many interpreters and compilers will signal coding problems upwards in the decision stack rather than blindly writing bad machine code instructions. As a result, the arrows of information flow are bi-directional in the decision stack, as with biological brains.

## 6. Explaining AI Failures: Towards an AI Decision-Stack

The challenge in providing such an explanation for AI lies in the distributed nature of virtually all ML systems. A paradigmatic example can be found in artificial neural networks where computational units, “neurons,” encode a decision in their activity pattern as influenced by the weight of their respective synaptic inputs [108]. As with biological neurons in brains, the size and complexity of the network conceal which members are the “key players” in any given decision. Similar to neural systems, there are no “grandmother” cells (In neuroscience, a “grandmother cell” is a theoretical construct of the single neuron that encodes the memory of your own grandmother. Evidence suggests that grandmother cells do not exist in complex biological brains.) [109].

**Figure 4:**
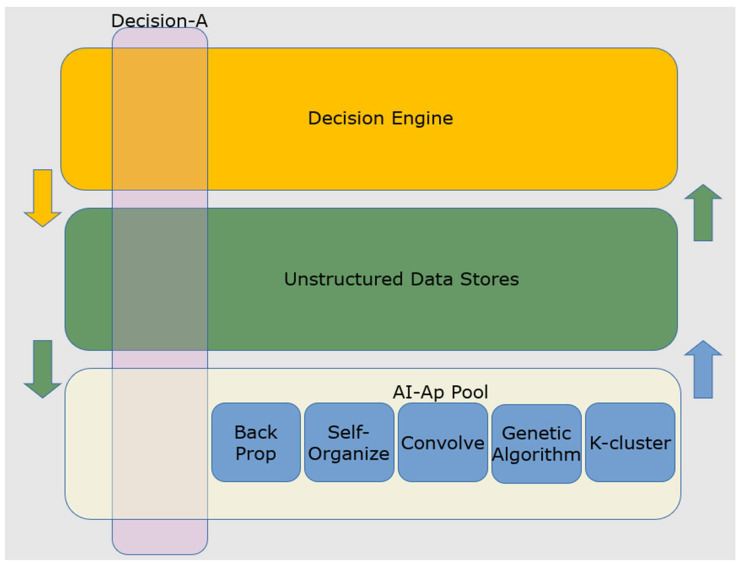
The decision stack for an AI decision machine. Pictured above is a schematized reference architecture upon which an AI decision stack (translucent pink) must operate. At the base are homogenous or heterogeneous populations of ML algorithms (AI-Ap Pool) that run on unstructured data. The examples in the figure include back propagation artificial neural networks, self-organizing networks, convolution algorithms, genetic algorithms, and K-clustering statistical methods. Datastores are implemented at the middle level. Such datastores, physically distributed, are accessed at the base of the reference architecture and the top as schematized by the arrows. At the top level is a decision engine that acts as a read-out of the entire decision stack. The decision engine acts as a read-out and an integral component of the decision stack that may implement some machine learning.

Additional complexity in such networks comes from the reference architecture of many AI’s where heterogeneous algorithms, unstructured data storage, and a separate decision engine resides. This is schematically represented in Figure 4.

As with biological nervous systems, the basis for a decision may be embedded in the instantiated ML networks at the base of Figure 4. It then propagates to shape the response of the top level: an AI-decision stack. The bottom layer of the stack in Figure 4 does not represent CMOS hardware (e.g., transistors). Instead, it represents the software-defined nodes (e.g., artificial neurons) of one or several ML algorithms. In this case, it should be possible to probe the universe of these nodes for their activity during a critical decision-making process, logging that data for future analysis in a manner analogous to the neurobiology experiments described above.

Further, and crucial to explainable AI, once such an “engram” is revealed, it should be possible, in a manner analogous to biological brains, to turn off the labeled nodes and to test whether the AI’s “decision” is reversed, as propagated across the AI-decision stack. Under our operational definition of explainable AI, the results of this test constitute *the explanation*, once again taking from the field of neuroscience.

## 7. The Neurally inspired Framework

In biological brains, an *explanation* for a single biological memory has been achieved by labeling the members of a cell assembly that, by the act of firing action potentials together, are functionally bound during memory formation (i.e., an engram). Then, by onpogenetically inactivating those cells, and only those cells, it is possible to reversibly control the recall of the specific memory [9]. We treat the mnemonic function as a specific instance of decision-making since each decision requires a corresponding memory. We introduce the notion of the decision stack, a biological “reference architecture.” The members of the cell assembly are in the lowest layer of this reference architecture.

In the case of our AI framework, we describe an analogous decision stack reference architecture where the individual nodes/neurons are also at the lowest layer. The instrumentation of these nodes (analogous to functional labeling and optogenetic control) enables one to label the relevant members to test for explanation analogously. The test consists of re-running the decision with those nodes inactivated and revealing the dependence of the decision upon those specific nodes as they propagate their activity across the AI-decision stack.

It is essential to point out that, as with biological brains, individual nodes may participate in many separate decisions and that an individually flagged node may not be crucial to explaining a single decision.

## 8. Existing Explainable AI Methods and Our Framework

AI-based systems allow powerful predictions. However, owing to their Blackbox nature, they are not readily explainable. Existing X-AI methods make a genuine effort to break the Blackbox yet cannot fully explain all the contours of a prediction [75,110,111]. As opposed to existing X-AI methods, our decision stack framework mimics biological brain principles. The natural existence of functionally-bound neurons phylogenetically conserved across all biological brains coupled with experimentally-induced optogenetic inactivation of ‘memory’ cells—leading to reversable memory recall—provides an empirical basis and existence proof for our decision stacks framework. While we have already pointed out challenges, including validity and explanation issues in current X-AI methods as well as laid the ground for comparison between existing X-AI methods and our framework in Section 3, we briefly make a few more remarks here.

Applying alternative X-AI explanation methods to similar input data may lead to different results [112]. This makes comparing results from different X-AI methods a daunting task. In other words, if we do not possess prior knowledge or background information on the data producers, we are unsure which X-AI explanation is more accurate. Our framework, taking inspiration from biological brain architectures, allows for a more plausible explanation. We foresee this explanation as objective (more precise and natural) and consistent (same result for similar input data).

Existing X-AI methods also present limitations based on the data type being used [113]. Data, as we know, come in various forms: numerical, image, text, and tabular. Often such data are unstructured. In a consequential environment where users and policymakers want an explanation, a powerful X-AI method should be well-equipped to handle any kind or combination of the data. However, the existing algorithms in X-AI methods seem unprepared to handle all data types [113]. Since we base our proposed framework on biology, which can process any data to make decisions, we foresee the algorithm in our framework as advantageous.

Finally, all X-AI methods face multifaceted challenges, as extensively elaborated in a recent article [75]. Some of these challenges pertain to the dynamics of data and decisions (changing data and decisions cause different explanations) and context dependency (since outcomes may differ for individuals, general explanations for algorithms may not work). Other challenges relate to the wicked nature of the problems addressed (due to the ambiguous and poorly structured nature of the problems, the problems could warrant multiple answers as opposed to a single answer provided by current algorithms) and contested explanations (explanations could be biased, for instance). While we do not think that our framework solves all these problems, we believe some of these problems (such as contested explanations, dynamic data, and context dependency) will likely be mitigated by the decision stacks framework that is inspired by actual biological mnemonic function in its design to return an explanation.

## 9. Conclusions

As the adoption of AI and ML continues to rise and reaches new audiences, increasingly complex Blackbox models (such as deep neural networks) pose explainability challenges to engineers, researchers, and policymakers alike. The future of AI’s use in consequential applications in health, justice, industry, defense, and security will increasingly require explainability. After summarizing the existing X-AI methods and some of their inherent issues in this paper, we theoretically propose using an AI-decision stack framework to operationalize AI explainability analogous to modern neuroscience. The operationalization would entail instrumenting the complete ML node set and recording them all during AI decision-making. The active nodes in this process define the test set for the explanation. A successful test requires demonstrating a causal relationship between the activation of the labeled nodes and the decision.

While in this paper, we aimed to furnish a theoretical framework, future research will allow for empirical testing of this framework on actual data. In principle, X-AI methods and frameworks bridge ML systems and human systems; thus, the decision stack framework testing and further refining will need collaborations between scholars in computer science, mathematics, economics, neuroscience, behavioral psychology, public policy, and experts in human-computer interaction.
